# Long-Term Reassurance with Negative High-Risk Human Papillomavirus (HR-HPV) and Clear Margins After Large Loop Excision of the Transformation Zone (LLETZ)

**DOI:** 10.3390/cancers17030487

**Published:** 2025-02-01

**Authors:** Fatima Heydari, Silvia de Sanjosé, Judith Peñafiel Muñoz, Maria-Eulalia Fernández-Montolí

**Affiliations:** 1 Medicine and Translational Research Doctorate Program, Faculty of Medicine and Health Sciences, Universitat de Barcelona, 08907 Barcelona, Spain; fheydahe7@alumnes.ub.edu; 2 Barcelona Institute for Global Health (ISGlobal), Campus Clinic, 08036 Barcelona, Spain; desanjose.silvia@gmail.com; 3 Cancer Epidemiology Research Program, Catalan Institute of Oncology (ICO), Bellvitge Biomedical Research Institute (IDIBELL), L’Hospitalet de Llobregat, 08908 Barcelona, Spain; jpenafiel@idibell.cat; 4 Gynecology Department, Hospital Universitari de Bellvitge, Bellvitge Biomedical Research Institute (IDIBELL), Universitat de Barcelona, L’Hospitalet de Llobregat, 08907 Barcelona, Spain

**Keywords:** cervical intraepithelial neoplasia, CIN2-3, LLETZ, HR-HPV, margin status, type of LLETZ excision, cone dimensions, conization length

## Abstract

Background/Objective: Women treated with large loop excision of the transformation zone (LLETZ) for cervical intraepithelial neoplasia grade 2-3 (CIN2-3) remain at risk of CIN2-3 and cervical cancer for many years. We assessed the roles of high-risk human papillomavirus (HR-HPV) post-LLETZ, surgical margins, and LLETZ characteristics on the long-term risk of CIN2-3. Methods: A retrospective observational study was performed using data for 432 women with a histological diagnosis of CIN2-3 treated by LLETZ between 1996 and 2020 and followed-up until October 2021 at Hospital Bellvitge in Barcelona, Spain. Age, surgical margins, 6-month HR-HPV status, excision type, and cone volume/dimensions were analyzed in association with the risk of persistent/recurrent CIN2-3. The cumulative probability of persistent/recurrent CIN2-3 was calculated using the Kaplan–Meier and Cox models. Results: Persistent/recurrent CIN2-3 was detected in 7.4%, with over 90% found within 5 years post-LLETZ. Predictors of persistent/recurrent CIN2-3 were HR-HPV (HR = 7.36, 95% CI = 3.55–15.26), involved margins (HR = 3.94, 95% CI = 1.68–9.25), uncertain margins (HR = 4.42, 95% CI = 1.55–12.55), and age ≥ 35 years (HR = 2.92, 95% CI = 1.19–7.13). Type 3 excision (*p* = 0.035) and cone length (*p* = 0.010) correlated with clear margins. The negative predictive value (NPV) of both negative HR-HPV and clear margins post-LLETZ was 98.7%. Conclusions: The combination of negative HR-HPV and clear margins post-LLETZ provides stronger reassurance against the risk of persistent/recurrent CIN2-3 than do LLETZ characteristics. However, larger excisions in older women likely reduce the risk of involved margins. Close surveillance, including repeat HR-HPV testing in the first 5 years post-LLETZ, is crucial.

## Simple Summary

Cervical intraepithelial neoplasia grade 2-3 (CIN2-3) is often treated by large loop excision of the transformation zone (LLETZ) to prevent invasive cervical cancer. However, there is a high risk of persistent/recurrent CIN2-3 and cervical cancer for up to 20 years post-treatment. Factors such as high-risk human papillomavirus (HR-HPV) and surgical margins influence this risk. Clinicians using LLETZ aim to achieve clear surgical margins while minimizing the volume of tissue removal and side effects. This retrospective study assessed the roles of HR-HPV status post-LLETZ, surgical margins, and LLETZ characteristics (excision type, cone volume, and dimensions [length, thickness, and circumference]) for long-term reassurance against persistent/recurrent CIN2-3 in a large population spanning 25 years. We showed greater reassurance associated with negative HR-HPV post-LLETZ and clear surgical margins, but not with LLETZ characteristics, although cone length and type 3 excision correlated with clear margins. These findings provide valuable insights into long-term outcomes that may help optimize LLETZ.

## 1. Introduction

Cervical cancer, the third leading cause of female mortality, is preceded by cervical intraepithelial neoplasia grade 3 (CIN3). Although cervical intraepithelial neoplasia grade 2 (CIN2) is a less reproducible indicator, it is generally considered a safe threshold for management [1,2]. According to The Lower Anogenital Squamous Terminology (LAST), CIN2 and CIN3 are collectively classified as high-grade squamous intraepithelial lesions (HSIL) [3]. Histologically confirmed CIN2-3 lesions are often treated with excisional procedures like the large loop excision of the transformation zone (LLETZ) or the loop electrosurgical excision procedure (LEEP), particularly in high-resource settings, to mitigate the risk of cervical cancer progression [4]. In addition, other techniques, including straight wire excision of the transformation zone (SWETZ) and needle excision of the transformation zone (NETZ), are commonly used in specialized centers [5,6]. Excisional treatment aims to remove the transformation zone (TZ) of the cervix and the squamocolumnar junction (SCJ), taking into consideration the lesion size, location, and patient age [7].

The 2011 International Federation of Cervical Pathology and Colposcopy (IFCPC) guidelines detail three excision types (1, 2, and 3) that align with transformation zone types 1 to 3. A type 3 excision can be performed with a single or double split (top-hat) procedure [8]. Moreover, the UK National Health Service (NHS) cervical screening program recommends excisional lengths of <10 mm for a type 1 excision, 10–15 mm for a type 2 excision, and 15–25 mm for a type 3 excision [9].

Despite initial excisional treatment, 4% to 18% of immunocompetent patients will go on to experience persistent or recurrent CIN2-3. Persistent lesions usually suggest undertreatment and often occur in involved margins within 2 years post-treatment [10,11]. CIN2-3 lesions, or even cervical cancer, can be detected as late as 20 years post-treatment, possibly arising from new lesions or slow progression [2,12]. Long-term follow-up studies often assess cervical cancer risk rather than CIN2-3 outcomes post-LLETZ [13,14]. In a previous study of 242 patients with CIN2-3 treated with LLETZ and followed for up to 20 years, we identified that high-risk human papillomavirus (HR-HPV) status and involved margins represented significant predictors of persistent/recurrent CIN2-3 [15]. Clinicians should aim for clear margins, seeking to balance the tissue volume removed and the potential side effects while minimizing the risk of new or recurrent precancerous lesions [16]. Further research is needed to compare the long-term role of HR-HPV status, margins, and LLETZ characteristics in the risk of new CIN2-3. Identifying factors associated with reduced risk could provide routes to lowering patient anxiety and the medical burden.

We aimed to reassess the association of HR-HPV status and margin status, while also examining the impact of excision types (1, 2, or 3), and cone volume/dimensions (length, thickness, and circumference) with persistent/recurrent CIN2-3 post-LLETZ in a larger series spanning 25 years, compared with our previous report [15].

## 2. Materials and Methods

### 2.1. Study Design

**Figure 1:**
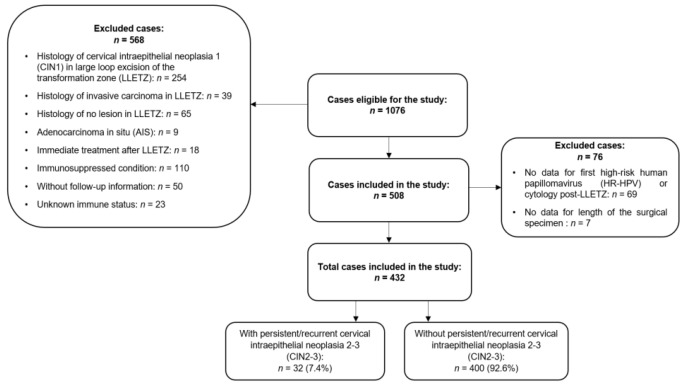
Flow chart of 1076 adult women who underwent large loop excision of the transformation zone (LLETZ) between 1996 and 2020 at Department of Gynecology in Bellvitge University Hospital. In total, 644 cases were excluded and 432 cases treated with LLETZ for cervical intraepithelial neoplasia 2-3 (CIN2-3) were included.

This retrospective observational study included women with a diagnosis of CIN who underwent LLETZ at the Department of Gynecology at Hospital Bellvitge in Barcelona, Spain. The hospital is a regional reference center for cervical lesion treatment in the Baix Llobregat health district. Follow-up information was retrieved from the reviews of the hospital and primary healthcare records of all registered women. We initially enrolled 1076 adult women who had undergone LLETZ for low-grade cervical intraepithelial neoplasia (CIN1), CIN2-3, adenocarcinoma in situ (AIS), cervical squamous cell carcinoma, and other cervical pathologies, including endocervical or ectocervical polyp and cervical nabothian cysts, between June 1996 and December 2020. Follow-up data collection ended in October 2021, giving a maximum follow-up period of 25 years. Ultimately, 432 (40%) women treated with LLETZ for a diagnosis of CIN2-3 with at least one follow-up visit (16.20%) post-LLETZ were included in the study (Figure 1). Prior to treatment, informed written consent was obtained from all patients. The project was approved by the research ethics committee. Figure 1 shows the inclusion and exclusion criteria of the study.

### 2.2. Surgical Procedure

All excisional procedures for primary CIN2-3 lesions were performed by LLETZ under paracervical local anesthesia. Lugol’s solution was applied to demarcate the area of abnormality, and the diathermic loop size was selected based on the size of the lesion. Endocervical curettage was not performed routinely after LLETZ; however, it was performed in very few cases (*n* = 31). Electrocoagulation was used to achieve hemostasis. The excised specimens were oriented anatomically with a stitch for pathological studies.

### 2.3. Type of Excision and Cone Volume/Dimensions

Data on the LLETZ excision types and dimensions were obtained from histology reports. After excision, the specimen was sent to the pathology unit for weighing and measurement to enable classification based on IFCPC and NHS criteria. The excision type was based on the transformation zone type, as per the 2011 IFCPC guidelines [8]. Length was the distance from the external to the internal margins, thickness was the distance from the stromal margins to the surface of the excised specimen, and circumference was the perimeter of the excised specimen [8].

Excision type categorization followed the 2016 NHS guidelines [9]. Cone volume was calculated using the Carcopino and Phadnis formulas. The Carcopino formula was “volume = (1/2) (4/3) × π × length × (circumference/2π) × thickness” [17]. The Phadnis formula was “volume = 1/2 (4/3) π (a/2) (b/2) c (where, a = transverse diameter, b = longitudinal diameter, c = depth)” [18].

### 2.4. Cytology

After LLETZ, patients originally underwent conventional cytology at 6 months, but this was later replaced by liquid-based cytology in 2012. In the conventional method, ectocervical and endocervical samples were obtained and cytology slides were stained using the Papanicolaou method. Liquid-based cytology used a cyto-brush for cell collection and the ThinPrep liquid-based medium for transport. The cytology findings were classified according to the 1989, 2001, or 2014 Bethesda system, depending on the year of analysis [19,20].

### 2.5. HR-HPV Determination

The presence of HR-HPV at 6 months post-LLETZ was tested by Hybrid Capture 2 (HC2) assay, with specimens collected using the Digene sample conversion kit (Digene, Gaithersburg, MD, USA). This assay is a signal-amplified hybridization antibody capture method that utilizes chemiluminescence to identify high-risk HPV types (i.e., 16, 18, 32, 34, 36, 39, 45, 51, 52, 56, 58, 59, 68). Chemiluminescence from the conjugated antibody-hybrid was measured by a luminometer in relative light units (RLUs). Samples were considered positive if the RLU was equal to or greater than the mean of a positive control (1.0 pg/mL) [21]. In 2019, the assay was replaced with the PCR Cobas^®^ 4800 HPV test that provided individual results for HPV-16 and HPV-18, as well as a grouped result for 12 other HR-HPV genotypes (31, 33, 35, 39, 45, 51, 52, 56, 58, 59, 66, and 68) in a single analysis [22].

### 2.6. Colposcopy

Colposcopy was conducted using a Carl Zeiss binocular colposcope (Jena, Germany) or an Optomic colposcope (Optomic, Madrid, Spain) and reported using the IFCPC terminology from 1990, 2002, and 2011 as appropriate [8]. During colposcopy, abnormal areas were visualized after applying 5% acetic acid or Lugol’s iodine solution. Endocervical curettage was performed for transformation zone type 3 or endocervical cases. Biopsies were taken for abnormal cytology results (e.g., atypical squamous cells of undetermined significance (ASCUS) or worse), positive HR-HPV tests post-LLETZ, or limited SCJ visualization.

### 2.7. Follow-Up

Follow-up appointments were set for 6 and 12 months post-LLETZ, including Pap smears and colposcopies. HR-HPV testing was performed at 6 months, and from approximately 2014 onward, was also performed at 12-months. If the surgical margins were positive, initial follow-up was scheduled at 3 months post-LLETZ and included HR-HPV testing, cytology, a colposcopy directed biopsy, and endocervical curettage. We excluded cases with involved margins who received immediate treatment after LLETZ. If biopsy confirmed CIN2-3, patients underwent repeat LLETZ; but, if the cervix was too short for a repeat LLETZ, they underwent hysterectomy. Patients returned to routine screening if they had a negative HR-HPV result post-LLETZ with two consecutive normal cytology results and normal colposcopies. From 2014 onward, routine screening was restarted if women had a negative HR-HPV result post-LLETZ followed by two consecutive negative co-tests at one- and three-year intervals.

### 2.8. Criteria for the Persistent/Recurrent CIN2-3

Persistent/recurrent CIN2-3 was defined as a cervical biopsy diagnosing CIN2-3 after an initial LLETZ treatment, necessitating repeat LLETZ or hysterectomy. CIN2 and CIN3 were analyzed together because we anticipated only a few cases. The detection of CIN2-3 during follow-up may suggest undertreatment, typically associated with persistent lesions identified within the first year post-LLETZ. CIN2-3 detected after the first year post-LLETZ was referred to as recurrent.

### 2.9. Statistical Methods

A custom electronic case report form was developed using Microsoft Access to facilitate prospective data entry. Data were extracted from electronic medical records retrospectively and retrieved into a Microsoft Excel database. Descriptive statistics included counts and percentages for categorical variables and the median (min–max) with interquartile range (IQR) for continuous variables. A two-sided *p*-value < 0.05 was considered to indicate a significant difference. Univariate and multivariate Cox proportional hazards models assessed associations with persistent/recurrent CIN2-3, and these are reported using hazard ratios (HR) and 95% confidence intervals (CI). The Chi-square test or Kruskal–Wallis test analyzed associations between surgical margins, excision type, and cone volume/dimensions. Multiple imputation by chained equations (MICE) addressed the missing data for type of excision. Treatment failure was calculated from the initial LLETZ to the detection of persistent/recurrent CIN2-3. The cumulative risk of persistent/recurrent CIN2-3 was estimated using the Kaplan–Meier approach. Statistical analysis was performed using R version 4.1.0 for Windows (R Core Team, 2021) and IBM SPSS version 25.0 (IBM Corp., 2017; Armonk, NY, USA).

## 3. Results

### 3.1. Study Population

From a total of 1076 women treated with LLETZ, 432 cases with CIN2-3 at baseline and follow-up information were included in the study extending the study follow-up from the year 2006 to 2021 [15]. Verification of the data until the year 2024 confirmed no new CIN2-3 detections during follow-up in recent LLETZ cases. Following the inclusion–exclusion criteria, the present study featured a cohort of 258 new cases compared to our earlier report [15]. Treatment success was observed in 400 (92.6%) cases and CIN2-3 was detected in 32 (7.4%) cases after the initial LLETZ procedure. Among the 32 CIN2-3 cases, histology reports indicated 3 cases of CIN2 (9.3%), 25 cases of CIN3 (78.1%), and 4 cases of CIN2-3 (12.5%).

### 3.2. Descriptive Characteristics of the Study Cohort

The median time for persistent/recurrent CIN2-3 was 11.5 months (interquartile range, 3.8–27.9). Persistent/recurrent CIN2-3 was diagnosed in 18 cases (56.2%) within the first 12 months post-LLETZ, with margins involved in 13 (72%) of these cases. Persistent/recurrent CIN2-3 was detected in 5 cases (15.6%) between 12 and 24 months post-LLETZ. A further 2 cases (6.2%) were diagnosed between 24 and 29 months, and 7 cases (21.9%) after 30 months. The longest time to detect CIN2-3 was 198.2 months post-LLETZ. We detected 14 (43.7%) CIN2-3 cases after 12 months post-LLETZ, with margins involved in 4 (28%) cases (3 endocervical and 1 ectocervical). In total, 90.6% of the CIN2-3 cases were detected within 5 years post-LLETZ. Among the 32 CIN2-3 cases detected during follow-up, 22 underwent repeat LLETZ (68.7%), 9 underwent hysterectomy (28.1%), and 1 was lost to follow-up (3.1%).

During the study, three cases of cervical cancer, one HPV-related vaginal cancer, and one HPV-related oropharyngeal cancer were detected. Furthermore, 65 cases (15%) were diagnosed with CIN1, of whom 31 (47.7%) received an LLETZ and 34 (52.3%) were monitored. One monitored case, a 52-year-old woman, developed CIN3 and then cervical cancer before undergoing hysterectomy.

Table 1 shows the characteristics of the 432 included women. The median follow-up time was 70.3 months (interquartile range, 17.9–141), with 75% of women followed for over 141 months. Post-treatment HR-HPV testing was positive in 100 women (23.1%), of whom 20 (20%) had a subsequent diagnosis of CIN2-3. Among 332 (76.9%) cases of negative HR-HPV post-LLETZ, only 12 (3.6%) cases experienced persistent/recurrent CIN2-3. The most delayed case was detected 143 months after treatment.

Table 2 shows the characteristics of surgical specimens. Surgical margins were involved in 157 (36.3%) cases, uncertain in 43 cases (10.0%), and clear in 232 cases (53.7%). Overall, the involved endocervical margins were more likely to be associated with a CIN2-3 diagnosis during follow-up than the absence of endocervical involvement (*p* < 0.01) (Table S1). The overall negative predictive values (NPVs) of HR-HPV and clear margins were 96.4% and 96.6%, respectively. For both combined, the NPV was 98.7% after follow-up.

### 3.3. Surgical Margins, Type of Excision, and Cone Volume/Dimensions

Table 3 shows the associations of surgical margins with age, type of excision, and cone volume/dimensions. Clear margins were more likely to be observed in women aged <35 years (61.9%) compared to women aged ≥35 years (47.8%) (*p* < 0.001). Excision type was also associated with margin status (*p*-value = 0.035). The proportion of clear margins was 46.1% for type 1 excisions, 53.8% for type 2 excisions, and 65.2% for type 3 excisions. However, there were no significant differences between excision type and either ectocervical or endocervical margin involvement (Table S2). Among the different cone dimensions, a longer surgical specimen length was significantly associated with clear margins (*p*-value = 0.010). The median lengths were 12 mm, 10 mm, and 8 mm for those with clear, involved, and uncertain margins, respectively.

### 3.4. Predictors of Persistent/Recurrent CIN2-3 by Multivariate Analysis

Table 4 shows the multivariate Cox regression analysis adjusted for the first HR-HPV positive result post-LLETZ, surgical margins, and age, including all variables associated with persistent/recurrent CIN2-3. In Figure S1, we observed that a positive HR-HPV result post-LLETZ, involved margins, and older age were associated with a higher risk of more frequent and earlier persistent/recurrent CIN2-3. In multivariate analysis, HR-HPV detection post-LLETZ was the strongest predictor of persistent/recurrent CIN2-3 compared with other factors (HR = 7.36, 95% CI = 3.55–15.26). There was an almost 4-fold increase in the HR among women with involved margins (HR = 3.9) or uncertain margins (HR = 4.4) compared with those who had clear margins. An age ≥ 35 years was associated with an HR of 2.9 compared with younger women. Adding treatment characteristics did not improve the models.

## 4. Discussion

### 4.1. Main Findings

Persistent/recurrent CIN2-3 occurred in 7.4% of the present cohort, compared with 5.7% in our previous report [15]. A 6-month negative HR-HPV test was associated with just 3.6% persistent/recurrent CIN2-3 cases at follow-up (1 case after 143 months), emphasizing the importance of negative HR-HPV test post-treatment. Adding margins as a predictive factor increased the NPV of 98.7%. After LLETZ, 56.2% of the CIN2-3 cases were detected within the first 12 months, likely indicating insufficient initial treatment, as 72% had margin involvement. The remaining CIN2-3 cases were detected after 12 months (besides 3 cases of cervical cancer). Over 90% of CIN2-3 cases were detected within 5 years post-LLETZ. Multivariate analysis showed that a positive HR-HPV result post-LLETZ, involved margins, uncertain margins, and age ≥ 35 years were significant predictors of subsequent CIN2-3, consistent with our previous findings [15]. The associations of excision type and cone dimensions with persistent/recurrent CIN2-3 were obscured by the effect of margin status, probably due to limited statistical power. However, as expected, the longest excision types, type 3 excisions and 12 mm lengths, were both associated with clear margins. Women aged ≥35 years were less likely to have clear margins. Overall, endocervical margin involvement confirmed the relevance of endocervical canal tissue in predicting further disease, consistent with other reports [10].

### 4.2. Strengths and Limitations

Our comprehensive search at the level of primary health, using our hospital’s main referral activities and an extended follow-up, provide deep insights into long-term lesion detection and cervical cancer risk. We investigated the joint effect of post-treatment HR-HPV results and surgical margins, a practical approach in prior studies. We also analyzed margin distribution across excision types and cone dimensions, performed volume comparisons, and conducted imputation strategies to minimize bias. However, the retrospective nature of our study limits the generalizability of our data. The presence of uncertain margins and wide 95% CIs in certain categories also posed challenges. Although our sample size was significantly increased compared with our previous study, we could still not avoid limitations based on the impact of LLETZ treatment characteristics on persistent/recurrent CIN2-3, because the sample size may have constrained the analysis of some hypotheses. Finally, the absence of type-specific HPV data made it difficult to distinguish between treatment failures and new cases, affecting the assessment of long-term cancer risk.

### 4.3. Interpretation

The presence of persistent/recurrent CIN2-3 cases in up to 7.4% of our cohort aligns with global average reports [10,23] and is lower than the 12.8% found in a recent study [24]. Contrary to our previous report and the existing literature [11,15,23], the present study spanning a median follow-up period of 70 months found that over 40% of CIN2-3 cases were diagnosed after the first year post-LLETZ, underscoring the benefit of active follow-up for 5 years.

The 2019 guidelines of the American Society for Colposcopy and Cervical Pathology (ASCCP) recommend HPV-based testing at 6 months post-treatment, annual testing until three negative results, and then surveillance every 3 years for 25 years [25]. Furthermore, the Spanish Association of Cervical Pathology and Colposcopy (AEPCC) suggest routine screening every 5 years after three negative co-tests following CIN2+ treatment [26]. These recommendations may reduce the risk of new CIN2-3 cases post-treatment. The elevated rate of CIN2-3 cases through the 5-year post-LLETZ period in our study may be attributed to irregular co-testing, which potentially contributed to an increased CIN2-3 detection rate at follow-up. Our results support a strict 5-year follow-up post-treatment. Despite the lack of regular co-testing, our study offers a basis for increased HR-HPV testing to compare outcomes during follow-up. Knowledge of the HPV type, along with regular HR-HPV testing, could provide improved treatment insights.

The current study, which expanded our original cohort almost two-fold [15], reaffirmed the original predictive factors for persistent/recurrent CIN2-3. While HR-HPV is the strongest risk factor, age and margins added predictive value. We also observed that women with both a positive HR-HPV result and involved margins had earlier and higher rates of persistent/recurrent CIN2-3, which is again consistent with the findings of our prior report. This emphasizes the need for personalized management based on HR-HPV and margin statuses [15]. Furthermore, in the current study we observed that a negative HR-HPV at 6-months post-LLETZ combined with clear margins provided greater reassurance than any LLETZ characteristics for predicting persistent/recurrent CIN2-3 over the long 25-year follow-up period.

Excision type, which was not examined in our previous report, did not affect persistent/recurrent CIN2-3 during follow-up; moreover, contrary to previous reports, we found no correlation between cone length and persistent/recurrent CIN2-3 [27]. A recent study by Foggiatto et al. noted that an excised endocervical canal length under 1.25 cm increased recurrence rates by 2.5 times [24].In our study, a low incidence of persistent/recurrent CIN2-3 post-LLETZ precluded drawing definitive conclusions. However, we found that 12 mm length was associated with clear margins. Compared with the study by Foggiatto et al., we had a higher rate of margin involvement; however, Foggiatto et al. observed more endocervical margin involvement, likely due to the shorter removed canal length that is associated with recurrence.

Despite our prior report, we examined the margin distribution across types of excision and cone volume/dimensions. We showed that type 3 excision in particular was significantly associated with clearer margins. This is in line with a recent study showing that negative endocervical margins were observed in 86% of type 2 excisions compared with 78% of type 1 excisions (type 3 excisions were not assessed) [28]. We observed no differences in ectocervical or endocervical margin involvement across various excision types, probably due to the very low numbers with either involvement in each excision type.

Additionally, our study suggests a length of 12 mm as a safe oncological limit for achieving clear margins, again consistent with previous reports [16,29]. Lengths over 10 mm may increase the risk of preterm delivery [30]. Women aged ≥35 years in the present study had a lower probability of clear margins and a greater risk of persistent/recurrent CIN2-3. Prioritizing a greater length, particularly type 3 excisions, is crucial for obtaining clear margins in older women, but risks cervical stenosis [31]. Moreover, some studies show a minor decline in sexual satisfaction after LLETZ, while others not [32,33]. The inward shift of the SCJ during the perimenopause and menopause reduces its visibility as it becomes positioned deeper within the endocervical canal. This shift is associated with a higher frequency of endocervical margin involvement and necessitates deeper excisions in older women; however, precancers may remain, contributing to persistent/recurrent CIN2-3 [34]. Further research is therefore needed to improve treatment options when the SCJ is not fully visible, ensuring that deeper lesions are addressed in surgery without unnecessary removal of stroma to avoid further side effects [34]. Furthermore, our recent understanding is that the islands of reserve cells may remain in the endocervical canal giving rise to potential dysplastic lesions [35].

In contrast to our prior study, we used two formulas to calculate cone volume [15]; however, consistent with other findings, we found no correlation between volume and persistent/recurrent CIN2-3 or margin status [36]. Length may be more influential for clearing endocervical glands, which can be as deep as 5.22 mm from the surface of the cervix [9]. Thus, increasing tissue volume will not guarantee lesion removal from these glands.

## 5. Conclusions

A negative HR-HPV status post-LLETZ in the presence of clear margins provides long-term reassurance when predicting persistent/recurrent CIN2-3. Tailoring excision type and length, especially in women aged ≥35 years, can reduce margin involvement and optimize patient outcomes. Strict follow-up for 5 years post-LLETZ remains necessary and should include periodic HR-HPV testing.

## Figures and Tables

**Table 1:** Follow-up time and patient characteristics of women treated with a large loop excision of the transformation zone (LLETZ) for cervical intraepithelial neoplasia 2-3 (CIN2-3).

Patient Characteristics	Total *n* (%) *	No Persistent/Recurrent CIN2-3 (%) **	Persistent/Recurrent CIN2-3 (%) **	*p*-Value
**Follow-up time (months)**				**0.000 ^#^**
Median (IQR)	70.9 (17.9–141)	87.7 (23–143.5)	11.5 (3.8–27.9)	
**Age (years)**				0.100 ^#^
Median (min–max)	36.1 (18.3–77)	36 (18.3–77)	39 (24–62)	
**Age at the time of LLETZ (years)**				**0.022 ^##^**
<35 years old	181 (41.9)	174 (96.1)	7 (3.9)	
≥35 years old	251 (58.1)	226 (90.1)	25 (9.9)	
**Smoking**				0.788 ^##^
No	195 (45.1)	181 (92.8)	14 (7.2)	
Yes	209 (48.4)	194 (92.8)	15 (7.2)	
Unknown	28 (6.5)	25 (89.3)	3 (10.7)	
**Parity**				0.063 ^##^
Nulliparous	118 (27.3)	111 (94.1)	7 (5.9)	
<4 full-term births	264 (61.1)	246 (93.2)	18 (6.8)	
≥4 full-term births	15 (3.5)	11 (73.3)	4 (26.7)	
Unknown	35 (8.1)	32 (91.4)	3 (8.6)	
**Contraceptive method**				0.824 ^##^
None	77 (17.8)	70 (90.9)	7 (9.1)	
Hormonal	113 (26.2)	103 (91.2)	10 (8.8)	
IUD	47 (10.9)	44 (93.6)	3 (6.4)	
Condoms	130 (30.1)	123 (94.6)	7 (5.4)	
Others	19 (4.4)	17 (89.5)	2 (10.5)	
Unknown	46 (106)	43 (93.5)	3 (6.5)	
**HPV vaccine pre- or post-LLETZ**				0.988 ^##^
No	326 (75.5)	302 (92.6)	24 (7.4)	
Yes	91 (21.1)	84 (92.3)	7 (7.7)	
Missing	15 (3.5)	14 (93.3)	1 (6.7)	
**HR-HPV result (HC2 and Cobas 4800)**				
**First HR-HPV post-LLETZ**				**0.000 ^##^**
Negative	332 (76.9)	320 (96.4)	12 (3.6)	
Positive	100 (23.1)	80 (80.0)	20 (20.0)	
**First RLU HR-HPV post-LLETZ**				**0.000 ^#^**
Median (IQR)	0.2 (0.14–0.70)	0.2 (0.14–0.5)	2.25 (0.295–120.15)	
**First RLU HR-HPV post-LLETZ category**				**0.000 ^##^**
Negative	301 (69.7)	289 (72.3)	12 (37.5)	
1–100 pg/mL	60 (13.9)	53 (88.3)	7 (11.7)	
>100 pg/mL	27 (6.3)	18 (66.7)	9 (33.3)	
Unknown	44 (10.2)	40 (90.9)	4 (9.1)	
**Total**	432 (100.0)	400 (92.6)	32 (7.4)	

CIN, cervical intraepithelial neoplasia; IQR, interquartile range (25–75%); IUD, intrauterine device; HPV, human papillomavirus; LLETZ, large loop excision of the transformation zone; HR-HPV, high-risk human papillomavirus; RLU, relative light unit. Values in bold indicate significant differences between the study groups. * Column percentage; ** row percentage; ^#^ Kruskal–Wallis test *p*-value; ^##^ Fisher’s exact test *p*-value.

**Table 2:** Surgical specimen characteristics in women treated with a large loop excision of the transformation zone (LLETZ) for cervical intraepithelial neoplasia 2-3 (CIN2-3).

Surgical Specimen Characteristics	Total *n* (%) *	No Persistent/Recurrent CIN2-3 (%) **	Persistent/Recurrent CIN2-3 (%) **	*p*-Value
**Margin status**				**0.001 ^##^**
Clear	232 (53.7)	224 (96.6)	8 (3.4)	
Ecto+/endo−	68 (15.7)	65 (95.6)	3 (4.4)	
Ecto−/endo+	70 (16.2)	58 (82.9)	12 (17.1)	
Ecto+/endo+	11 (2.5)	9 (81.8)	2 (18.2)	
Endo+/deep+	2 (0.45)	2 (100.0)	0 (0.00)	
All	4 (0.9)	4 (100.0)	0 (0.00)	
Deep	2 (0.45)	2 (100.0)	0 (0.00)	
Uncertain	43 (10.0)	36 (83.7)	7 (16.3)	
**Margin status category**				**0.002 ^##^**
Clear	232 (53.7)	224 (96.6)	8 (3.4)	
Involved	157 (36.3)	140 (89.2)	17 (10.8)	
Uncertain	43 (10.0)	36 (83.7)	7 (16.3)	
**Type of excision**				0.678 ^###^
Type 1 ^1^	141 (32.6)	129 (91.5)	12 (8.5)	
Type 2 ^2^	199 (46.1)	184 (92.5)	15 (7.5)	
Type 3 ^3^	92 (21.3)	87 (94.6)	5 (5.4)	
**Length (mm) ^4^**				0.356 **^#^**
Median (IQR)	10.0 (7.0–10.5)	10.0 (7.0–16.0)	9.5 (7.0–13.0)	
**Thickness (mm) ^5^**				0.172 **^#^**
Median (IQR)	10.5 (9.5–12.5)	11.0 (9.0–13.0)	10.0 (9.0–13.0)	
**Circumference (mm) ^6^**				0.461 **^#^**
Median (IQR)	103.0 (90.6–120.0)	101.0 (89.0–120.0)	99.0 (85.5–119.0)	
**Volume Carcopino (cm^3^) ^7^**				0.660 **^#^**
Median (IQR)	3.94 (2.27–6.18)	3.93 (2.27–6.17)	3.66 (1.60–7.24)	
**Volume Phadnis (cm^3^) ^8^**				0.329 **^#^**
Median (IQR)	2.18 (1.31–3.50)	2.26 (1.31–3.54)	1.96 (1.19–2.93)	
**Number of quadrants involved**				0.158 ^##^
1–2	203 (47.0)	190 (93.6)	13 (6.4)	
3–4	158 (36.6)	142 (89.9)	16 (10.1)	
Not evaluable	18 (4.2)	16 (88.9)	2 (11.1)	
Unknown	53 (12.3)	52 (98.1)	1 (1.9)	
**Total**	432 (100.0)	400 (92.6)	32 (7.4)	

CIN, cervical intraepithelial neoplasia; Ecto, ectocervical; Endo, endocervical. ^1^ Length of <10 mm. ^2^ Length of 10–15 mm. ^3^ Length of 15–25 mm. ^4^ The distance from the external margins to the internal margins, with data available from 307 cases for inclusion in the analysis. IQR, interquartile range (25–75%). ^5^ The distance from the stromal margins to the surface of the excised specimen. ^6^ The perimeter of the excised specimen, formula: 2 × 3.14159 × sqrt((cone amplitude^2^ + cone depth^2^)/2), with data available from 363 cases for inclusion in the analysis. ^7^ Volume = (1/2) (4/3) π × length × (circumference/2π) × thickness, with data available from 305 cases for inclusion in the analysis. ^8^ Volume = 1/2 (4/3) π (a/2) (b/2) c [a: transverse diameter, b: longitudinal diameter, c: depth], with data available from 302 cases for inclusion in the analysis. Values in bold indicate significant differences between the study groups. * Column percentage; ** row percentage; ^#^ Kruskal–Wallis test *p*-value; ^##^ Fisher’s exact test *p*-value; ^###^ Chi-square test *p*-value.

**Table 3:** Association of age, type of excision, and cone volume/dimensions with the margin status in women treated with a large loop excision of the transformation zone (LLETZ) for cervical intraepithelial neoplasia 2-3 (CIN2-3).

Variables	*n* = 432	Clear Margins	Involved Margins	Uncertain Margins	*p*-Value
**Age (years)**					**<0.001 ^#^**
Median (min–max)		35 (18–66)	36 (20–77)	42 (22–69)	
**Age (years)**					**<0.001^###^**
<35 years old, n (%) *	181	112 (61.9)	62 (34.3)	7 (3.9)	
≥35 years old, n (%) *	251	120 (47.8)	95 (37.8)	36 (14.3)	
**Type of excision**					**0.035 ^###^**
Type 1, n (%) *^1^	141	65 (46.1)	56 (39.7)	20 (14.2)	
Type 2, n (%) *^2^	199	107 (53.8)	76 (38.2)	16 (8.0)	
Type 3, n (%) *^3^	92	60 (65.2)	25 (27.2)	7 (7.6)	
**Length (mm) ^4^**					**0.010 ^#^**
Median (IQR)	307	12.0 (8.0–16.0)	10.0 (7.0–13.7)	8.0 (5.0–13.0)	
**Thickness (mm) ^5^**					0.742 ^#^
Median (IQR)	432	10.5 (9.0–12.5)	10.5 (9.5–12.5)	11.0 (10.0–12.5)	
**Circumference (mm) ^6^**					0.760 ^#^
Median (IQR)	363	105 (88.9–120)	100 (90.2–120)	100 (94.1–120)	
**Volume Carcopino (cm^3^) ^7^**					0.524 ^#^
Median (IQR)	305	3.97 (2.42–6.13)	3.85 (1.95–6.41)	3.69 (1.53–7.09)	
**Volume Phadnis (cm^3^) ^8^**					0.347 ^#^
Median (IQR)	302	2.35 (1.42–3.49)	2.09 (1.18–3.38)	2.15 (0.92–3.68)	
**Total**	432	232	157	43	

This table used the available data for each variable. ^1^ Length of <10 mm. ^2^ Length of 10–15 mm. ^3^ Length of 15–25 mm. ^4^ The distance from the external margins to the internal margins. IQR, interquartile range (25–75%). ^5^ The distance from the stromal margins to the surface of the excised specimen. ^6^ The perimeter of the excised specimen, formula: 2 × 3.14159 × sqrt((cone amplitude^2^ + cone depth^2^)/2). ^7^ Volume = (1/2) (4/3) π × length × (circumference/2π) × thickness. ^8^ Volume = 1/2 (4/3) π (a/2) (b/2) c [a: transverse diameter, b: longitudinal diameter, c: depth]. Values in bold indicate significant differences between the study groups. * Row percentage; ^#^ Kruskal–Wallis test *p*-value; ^###^ Chi-square test *p*-value.

**Table 4:** Multivariate analysis of the significant factors associated with persistent/recurrent CIN2-3 after large loop excision of the transformation zone (LLETZ) in women with CIN2-3.

Variables	HR (95% CI)	*p*-Value ^a^
HR-HPV test post-LLETZ (negative vs. positive)	7.36 (3.55–15.26)	**<0.001**
Involved vs. clear margins	3.94 (1.68–9.25)	**0.002**
Uncertain vs. clear margins	4.42 (1.55–12.55)	**0.005**
age ≥ 35 years old vs. <35 years old	2.92 (1.19–7.13)	**0.019**

CI, confidence interval; HR, hazard ratio; HR-HPV, high-risk human papillomavirus; LLETZ, large loop excision of the transformation zone. Values in bold indicate significant differences between the study groups. ^a^ Log likelihood-ratio test *p*-value was used for this column.

## Data Availability

The data presented in this study are available on request from the corresponding author. The data are not publicly available due to the location of the database in the intranet of Bellvitge Hospital.

## Supplementary Materials

The following supporting information can be downloaded at: https://www.mdpi.com/article/10.3390/cancers17030487/s1, Figure S1: Kaplan–Meier curves for persistent/recurrent CIN2-3 (A) by HR-HPV post-LLETZ (*p* log-rank < 0.001), (B) by margin status (*p* log-rank < 0.001), (C) by margin status for positive HR-HPV post-LLETZ (*p* log-rank < 0.001), (D) by margin status for negative HR-HPV post-LLETZ (*p* log-rank = 0.122), (E) by age (*p* log-rank = 0.013), (F) by age for positive HR-HPV post-LLETZ (*p* log-rank < 0.001), (G) by age for negative HR-HPV post-LLETZ (*p* log-rank = 0.636). Table S1: Univariate analysis of predictive factors associated with persistent/recurrent CIN2-3 after large loop excision of the transformation zone (LLETZ) in women with CIN2-3. Table S2: Association between type of excision, cone length, age, and first HR-HPV post-LLETZ with ectocervical and endocervical margins of the surgical specimen after treatment with large loop excision of the transformation zone (LLETZ) for cervical intraepithelial neoplasia 2-3 (CIN2-3).
