# Supplementary material for: Long-Term Reassurance with Negative High-Risk Human Papillomavirus (HR-HPV) and Clear Margins After Large Loop Excision of the Transformation Zone (LLETZ)
Source: Cancers (Basel). 2025 Feb 1;17(3):487. doi: 10.3390/cancers17030487 (PMC11816363; doi:10.3390/cancers17030487)
Supplement: Supplementary file 1 [file cancers-17-00487-s001.zip › Supplementary Figure.pdf]

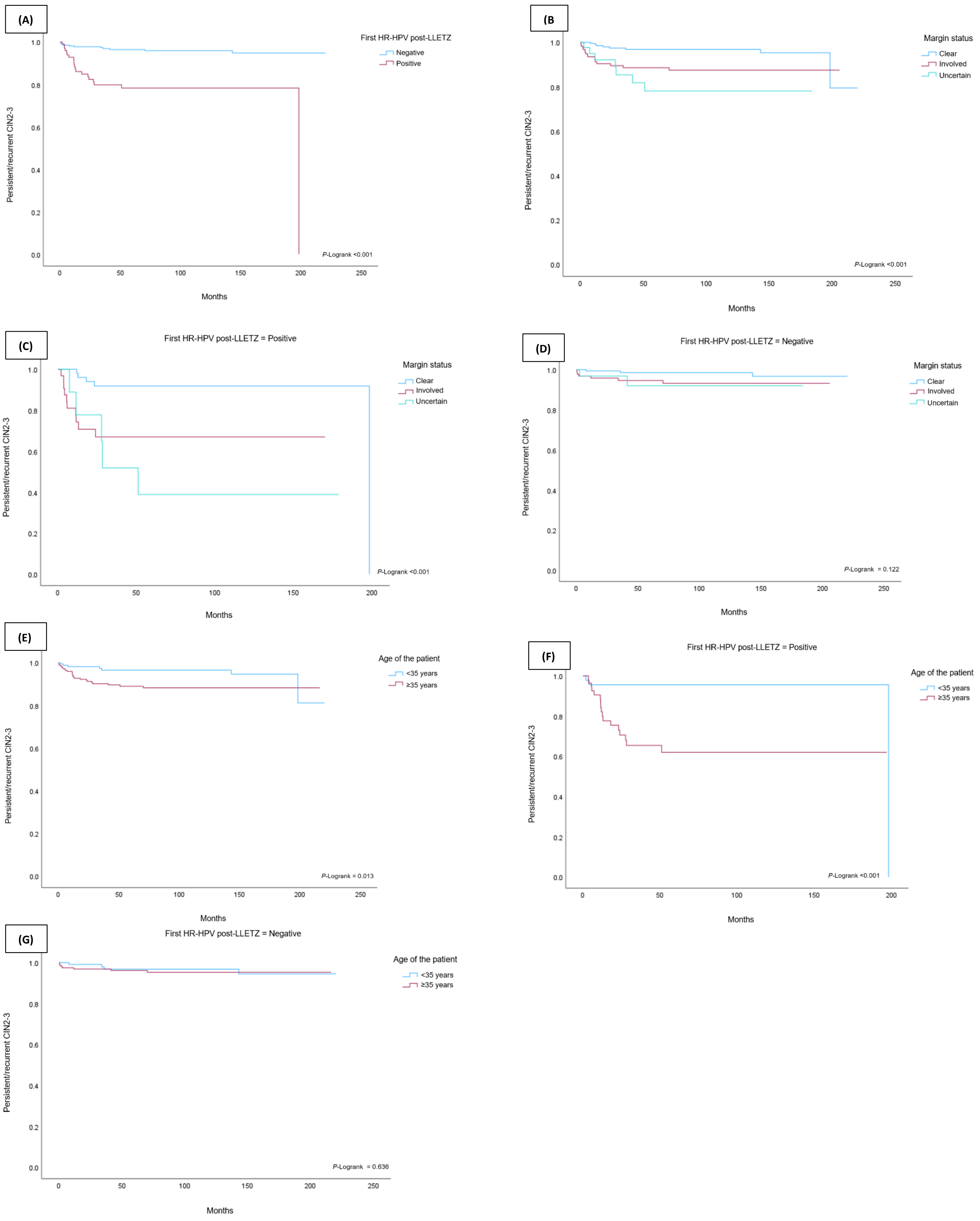
(A)
First HR-HPV post-LLETZ
Negative
Positive
Persistent/recurrent CIN2-3
1.0
0.8
0.6
0.4
0.2
0.0
0
50
100
150
200
250
Months
P-Logrank <0.001
(B
Margin status
Clear
Involved
Uncertain
Persistent/recurrent CIN2-3
1.0
0.8
0.6
0.4
0.2
0.0
0
50
100
150
200
250
Months
P-Logrank <0.001
(C)
First HR-HPV post-LLETZ = Positive
Margin status
Clear
Involved
Uncertain
Persistent/recurrent CIN2-3
1.0
0.8
0.6
0.4
0.2
0.0
0
50
100
150
200
Months
P-Logrank <0.001
(D)
First HR-HPV post-LLETZ = Negative
Margin status
Clear
Involved
Uncertain
Persistent/recurrent CIN2-3
1.0
0.8
0.6
0.4
0.2
0.0
0
50
100
150
200
250
Months
P-Logrank = 0.122
(E)
Age of the patient
<35 years
≥35 years
Persistent/recurrent CIN2-3
1.0
0.8
0.6
0.4
0.2
0.0
0
50
100
150
200
250
Months
P-Logrank = 0.013
(F)
First HR-HPV post-LLETZ = Positive
Age of the patient
<35 years
≥35 years
Persistent/recurrent CIN2-3
1.0
0.8
0.6
0.4
0.2
0.0
0
50
100
150
200
Months
P-Logrank <0.001
(G)
First HR-HPV post-LLETZ = Negative
Age of the patient
<35 years
≥35 years
Persistent/recurrent CIN2-3
1.0
0.8
0.6
0.4
0.2
0.0
0
50
100
150
200
250
Months
P-Logrank = 0.636

Figure S1. Kaplan-Meier curves for persistent/recurrent CIN2-3 (A) by HR-HPV post-LLETZ (p log-rank < 0.001), (B) by margin status (p log-rank < 0.001), (C) by margin status for positive HR-HPV post-LLETZ (p log-rank < 0.001), (D) by margin status for negative HR-HPV post-LLETZ (p log-rank = 0.122), (E) by age (p log-rank = 0.013), (F) by age for positive HR-HPV post-LLETZ (p log-rank < 0.001), (G) by age for negative HR-HPV post-LLETZ (p log-rank = 0.636).
