# Supplementary material for: Long-Term Reassurance with Negative High-Risk Human Papillomavirus (HR-HPV) and Clear Margins After Large Loop Excision of the Transformation Zone (LLETZ)
Source: Cancers (Basel). 2025 Feb 1;17(3):487. doi: 10.3390/cancers17030487 (PMC11816363; doi:10.3390/cancers17030487)
Supplement: Supplementary file 1 [file cancers-17-00487-s001.zip › Supplementary Tables.pdf]

Table S1. Univariate analysis of predictive factors associated with persistent/recurrent CIN2-3 after large loop excision of the transformation zone (LLETZ) in women with CIN2-3.

Characteristics	Total n	CIN2-3 n (%)	HR	95% CI	P-value[a]
**Age at the time of LLETZ (years)**					
< 35 years old	181	7 (3.8)	Ref.		Ref.
≥ 35 years old	251	25 (9.9)	2.84	(1.20-6.70)	**0.017**
**Age (years)**					
Median (min-max)	36.1 (18.3-77)	39.2 (24-62)	1.02	(0.99-1.06)	0.184
**Smoking**					
No	195	14 (7.2)	Ref.		Ref.
Yes	209	15 (7.2)	0.93	(0.45-1.93)	0.850
Unknown	28	3 (10.7)	1.51	(0.42-5.41)	0.526
**Parity**					
Nulliparous	118	7 (5.9)	Ref.		Ref.
< 4 full term births	264	18 (6.8)	1.08	(0.45-2.59)	0.862
≥ 4 full-term births	15	4 (26.7)	4.99	(1.46-17.1)	**0.010**
Unknown	35	3 (8.6)	1.19	(0.30-4.75)	0.802
**Contraceptive method**					
Nothing	77	7 (9.1)	Ref.		Ref.
Hormonal	113	10 (8.8)	0.59	(0.20-1.68)	0.321
IUD	47	3 (6.4)	1.04	(0.39-2.72)	0.944
Condom	130	7 (5.4)	0.60	(0.15-2.38)	0.471
Others	19	2 (10.5)	1.09	(0.22-5.43)	0.916
**HPV vaccine pre- or post-LLETZ**					
No	326	24 (7.4)	Ref.		Ref.
Yes	91	7 (7.7)	1.19	(0.51-2.78)	0.696
**HR-HPV result (HC2 and Cobas 4800)**					
**First HR-HPV post-LLETZ**					
Negative	332	12 (3.6)	Ref.		Ref.
Positive	100	20 (20.0)	5.98	(2.92-12.3)	**< 0.001**
**First RLU HR-HPV post-LLETZ**					
Median (Min-Max)	0.21 (0.06-3249.9)	0.23 (0.07-561.43)	1.00	(0.99-1.01)	0.604
**First RLU HR-HPV post-LLETZ category**					
Negative	301	12 (37.5)	Ref.		Ref.
1-100 pg/ml	60	7 (11.7)	2.86	(1.12-7.26)	**0.027**
> 100 pg/ml	27	9 (33.3)	11.0	(4.60-26.2)	**< 0.001**
Unknown	44	4 (9.1)	2.98	(0.93-9.56)	0.067
**Surgical specimen characteristics**					
**Margin status**					
Clear	232	8 (3.4)	Ref.		Ref.
Ecto+/endo-	68	3 (4.4)	1.43	(0.38-5.38)	0.600
Ecto-/endo+	70	12 (17.1)	5.64	(2.30-13.8)	**< 0.001**
Ecto+/endo+	11	2 (18.2)	6.86	(1.45-32.5)	**0.015**
Endo+/deep+	2	0 (0.00)*	0.00	(0.00)	0.999

All	4	0 (0.00)*	0.00	(0.00)	0.998

Table S1. (Continued)

Characteristics	Total n	CIN2-3 n (%)	HR	95% CI	P-value[a]
Deep	2	0 (0.00)*	0.00	(0.00)	0.999
Uncertain	43	7 (16.3)	5.54	(1.99-15.4)	**0.001**
**Margin status category**					
Clear	232	8 (3.4)	Ref.		Ref.
Involved	157	17 (10.8)	3.56	(1.53-8.25)	**0.003**
Uncertain	43	7 (16.3)	5.53	(1.99-15.4)	**0.001**
**Type of excision**					
Type 1[1]	141	12 (8.5)	Ref.		
Type 2[2]	199	15 (7.5)	0.92	(0.33-2.56)	0.540
Type 3[3]	92	5 (5.4)	0.71	(0.22-2.29)	0.272
**Length (mm)[4]**					
Median (IQR)	10.0 (7.0-15.0)	9.5 (7.0-13.0)	0.84	(0.37-1.94)	0.688
**Thickness (mm)[5]**					
Median (IQR)	10.5 (9.5-12.5)	10.0 (9.0-13.0)	0.34	(0.08-1.44)	0.143
**Circumference (mm)[6]**					
Median (IQR)	103.0 (90.6-120.0)	99.0 (85.5-119.0)	0.92	(0.78-1.10)	0.363
**Volume Carcopino (cm^3)[7]**					
Median (IQR)	3.94 (2.27-6.18)	3.66 (1.60-7.24)	0.98	(0.88-1.10)	0.761
**Volume Phadnis (cm^3)[8]**					
Median (IQR)	2.18 (1.31-3.50)	1.96 (1.19-2.93)	0.86	(0.65-1.12)	0.263
**Quadrants involved**					
1-2	203	13 (6.4)	Ref.		Ref.
3-4	158	16 (10.1)	0.54	(0.12-2.43)	0.429
Not valuable	18	2 (11.1)	0.93	(0.21-4.09)	0.933
Unknown	53	1 (1.9)	0.19	(0.01-2.18)	0.185
**Total**	432	32 (7.4)			

CI, confidence interval; HR, hazard ratio; LLETZ, large loop excision of the transformation zone; IUD, intrauterine device; HPV, human papilloma virus; HR-HPV, high-risk human papilloma virus; RLU, relative light unit; Ecto, ectocervical; Endo, endocervical; IQR, interquartile range (25-75%). [1]Length of <10 mm. [2]Length of 10-15 mm. [3]Length of 15-25 mm. [4]The distance from the external margins to the internal margins, with data available from 307 cases for inclusion in the analysis. [5]The distance from the stromal margins to the surface of the excised specimen. [6]The perimeter of the excised specimen, formula: 2*3.14159*sqrt((cone amplitude^2+cone depth^2)/2), with data available from 363 cases for inclusion in the analysis. [7]Volume = (1/2) (4/3) π × length × (circumference /2π) × thickness, with data available from 305 cases for inclusion in the analysis. [8]Volume = 1/2 (4/3) π (a/2) (b/2) c [a: transverse diameter, b: longitudinal diameter, c: depth] with data available from 302 cases for inclusion in the analysis. Values in bold indicate significant differences between study groups. *No recurrences observed in the category. [a]Log-likelihood ratio test p-value was used for this column.

Table S2. Association between type of excision, cone length, age, and first HR-HPV post-LLETZ with ectocervical and endocervical margins of the surgical specimen after treatment with large loop excision of the transformation zone (LLETZ) for cervical intraepithelial neoplasia 2-3 (CIN2-3).

	**n = 138****	**EctocervicaL margin involvement n= 68**	**EndocervicaL margin involvement n = 70**	**OR (95% CI)**	**P-value[a]**
**Type of excision**					
Type 1, n (%)*	46 (33.3)	23 (50.0)	23 (50.0)	Ref.	Ref.
Type 2, n (%)*	70 (50.7)	34 (48.6)	36 (51.4)	1.01 (0.44-2.32)	0.985
Type 3, n (%)*	22 (15.9)	11 (50.0)	11 (50.0)	0.98 (0.32-3.20)	0.979
**Length (cm)**					
Median (IQR)	1.00 (0.70-1.37)	1.00 (0.70-1.33)	1.00 (0.70-1.37)	1.29 (0.62-2.68)	0.496
**Age (years)**					
< 35 years old, n (%)*	53 (38.4)	30 (56.6)	23 (43.4)	Ref.	Ref.
≥ 35 years old, n (%)*	85 (61.6)	38 (44.7)	47 (55.3)	0.767 (0.445-1.320)	0.338
**First HR-HPV post-LLETZ**					
Negative, n (%)*	110 (79.7)	58 (52.7)	52 (47.3)	Ref.	Ref.
Positive, n (%)*	28 (20.3)	10 (35.7)	18 (64.3)	1.800 (0.831-3.899)	0.136

CI, confidence interval; OR odd ratio; IQR, interquartile range (25-75%); HR-HPV, high-risk human papilloma virus; LLETZ, large loop excision of the transformation zone; *row percentage; **Column percentage; [a]Log-likelihood ratio test p-value was used for this column.
